# The Gut Microbiome Profile of Lions in Etosha National Park, Namibia

**DOI:** 10.21203/rs.3.rs-9092464/v1

**Published:** 2026-05-06

**Authors:** Carl Belger, Jakob Wirbel, Dylan Maghini, Nadia Carstens, Ansia van Coller, James C. Beasley, Jörg Melzheimer, Aaron Y. Berkman, Willem Maartin Strauss, Robyn S. Hetem, Scott Hazelhurst

**Affiliations:** 1 School of Animal, Plant and Environmental Science, University of the Witwatersrand, Johannesburg, South Africa.; 2 Sydney Brenner Institute for Molecular Bioscience, University of the Witwatersrand, Johannesburg, South Africa.; 3 Division of Hematology, Department of Medicine, Stanford University, Stanford, CA, United States.; 4 Department of Human Genetics, Stanford University, Stanford, CA, United States.; 5 Genomics Platform, South African Medical Research Council, Cape Town, South Africa.; 6 Division of Human Genetics, National Health Laboratory Service and School of Pathology, Faculty of Health Sciences, University of the Witwatersrand, Johannesburg, South Africa.; 7 Savannah River Ecology Laboratory, Warnell School of Forestry and Natural Resources, University of Georgia, Aiken, South Carolina, United States of America.; 8 Department Evolutionary Ecology, Leibniz Institute for Zoo and Wildlife Research, Berlin, Germany.; 9 Applied Behavioural Ecology and Ecosystem Research Unit, Department of Environmental Science, University of South Africa, Johannesburg, South Africa.; 10 School of Biological Sciences, University of Canterbury, New Zealand.; 11 School of Electrical & Information Engineering, University of the Witwatersrand, Johannesburg, South Africa.

**Keywords:** Panthera Leo, microbiome, Namibia, conservation, metagenomics

## Abstract

**Background:**

The gut microbiome plays a crucial role in carnivore ecology, diet, and health, yet remains poorly characterised in African lions (*Panthera leo melanochaita*). Previous studies of lion microbiomes have primarily focused on small numbers of captive individuals maintained on controlled diets of Asian origin, reporting Fusobacteriota and Firmicutes as dominant phyla. Some recent literature has begun to describe microbiome composition in free-living African lions; however, genome-resolved analyses and detailed functional characterisation of the wild African lion gut microbiome remain lacking.

**Results:**

We present the first comprehensive gut microbiome analysis of free-living African lions, including novel MAGs generated from examining 23 fresh faecal samples from 20 individuals in Etosha National Park, Namibia. The African lion gut was dominated by Bacteroides (22.1%) and Phocaeicola (13.3%) - two related genera - contrasting sharply with the captive lions where Fusobacterium (Bhopal, India) and Firmicutes (Rotterdam, Netherlands) predominate. This divergence likely reflects dietary differences, captivity effects and possibly allopatric separation. While recent work has begun to characterise taxonomic composition in wild African lions, our study extends these findings through the reconstruction of 318 bacterial and 102 viral metagenome-assembled genomes (MAGs) from combined short- and long-read sequencing data. Most MAGs shared <95% average nucleotide identity with existing reference genomes, indicating largely novel species. Supplementing the GTDB database with these MAGs reduced unclassified reads from 24.5% to 9.2%, demonstrating the substantial gaps in existing carnivore gut microbiome databases. Functional analysis revealed metabolic pathway enrichment, particularly for purine metabolism—critical for processing the lions’ high-purine diet—with nearly complete pathways for degrading adenine and guanine to urea.

**Conclusions:**

This study provides the first in depth description of the microbial taxa in the African lion gut microbiome. Genera in the Bacteroidaceae family dominated. There are large differences with the metagenomics of the ***n* = 3,4** hybrid and Asiatic lions on controlled diets reported in prior studies. The discovery of over 300 novel MAGs significantly expands microbial reference databases and underscores the unique and understudied nature of apex carnivore microbiomes. These findings show critical microbial contributions to carnivore nutrition and establish a foundation for microbiome-based approaches to wildlife health monitoring and conservation management of threatened lion population.

## 1 Background

Microbial communities in the gut are linked to key health factors such as diet and disease (McKenney et al., 2018; Trevelline et al., 2019). The human (Almeida et al., 2019) and murine (Walter et al., 2020) gut microbiomes are arguably the most well understood of mammals while the microbiomes of wild animals, especially large carnivores, remain largely unexplored (Paleo-López et al., 2026). Characterising microbiomes of free-living mammals, particularly those species of conservation concern, may aid conservation efforts for a few reasons. Firstly, the gut microbiome is closely related to global environmental change and health (Bahrndorff et al., 2016; Trevelline et al., 2019; West et al., 2019). Secondly, new microbiology tools are increasingly being integrated into conservation efforts, such as faecal microbiome transplants (FMTs) (Guo et al., 2020; Bornbusch et al., 2024) and disease-marking bacterial species (Nkera-Gutabara et al., 2022). Studies have shown that gut microbiome diversity is reduced in species in fragmented and urbanised environments (Amato et al., 2013; Teyssier et al., 2018). However, physical interventions are uncommon because they can also result in harmful outcomes, especially when the microbiome associated with hosts is not understood for most species (Dallas and Warne, 2023). Therefore, creating microbiome catalogues is the first step to assessing the need for interventions.

Current research aims at uncovering the complex interplay between host health and microbiome composition, however our level of understanding varies greatly between different host species (Maritan et al., 2024). For example, the gut microbiomes of carnivores are not as well studied as those of herbivores and omnivores (Zoelzer et al., 2021). Yet, carnivores play a fundamental role in maintaining ecosystem function (Hoeks et al., 2020) and apex predators in particular are keystone species (Sergio et al., 2008), contributing to top-down regulation of trophic-levels (Lwin et al., 2025). Current research has identified the most abundant bacterial phyla in the gut of many carnivores as Bacillota, Fusobacteriota and Bacteroidota (de Jonge et al., 2022; Zhu et al., 2018). These phyla dominate the gut microbiome of carnivores in numerous studies to date, including spotted seals (*Phoca largha*), spotted hyena (*Crocuta crocuta*), Siberian tigers (*Panthera tigris altaica*), Bengal tigers (*Panthera tigris tigris*), jaguar (*Panthera onca*) and red foxes (*Vulpes vulpes*) (de Jonge et al., 2022; Zhu et al., 2018). Interestingly, strictly meat-eating carnivores show uniquely high proportions of Fusobacteriota compared to omnivores and herbivores, indicating that this phylum may be important to high protein and fat diets or that these diets may select for Fusobacteriota (Levin et al., 2021; Zhu et al., 2018). At a functional level, carnivores carry a relatively high proportion of genes involved in uric acid degradation in their gut microbiome (Zhu et al., 2018) facilitating the breakdown of the high purine diet of obligate carnivores.

Despite the vital role of the gut microbiome in carnivore health and ecology, there remains a notable scarcity of species-specific data, with most studies aggregating carnivores together (Wu et al., 2022; Youngblut et al., 2020; Zhu et al., 2018). As the apex predator of African savanna, insights into the microbiome of lions may improve assessments of ecosystem health and inform conservation management. There have been few lion gut microbiome studies. Mittal et al. (2020) compared the microbiome of three “African-Asian hybrid” lions (*Panthera leo*) and nine tigers (*Panthera tigris*) in at the Van Vihar National Park, India (Mittal et al., 2020). This park is ≈ 5km^2^ in area, and the carnivores have controlled diets. The microbiome of tigers and leopards were consistent with many other carnivores in having the highest abundance of Bacillota (32% and 40%, respectively), but the microbiome of these lions had Fusobacteriota as the most abundant phylum. A study of four Asiatic lions at the Rotterdam zoo ^1^, nine leopards (*Panthera pardus*) investigated the impact of changes in diet on the lions (Sun et al.): as expected diet had a significant impact on the microbiota which makes it likely that there will be significant differences with wild lions, which are also much more physically active.

This paper aims to provide a comprehensive analysis of the gut microbiome in free-living, African lions in Etosha National Park, Namibia (22000km^2^ in area).

## 2 Results

### 2.1 Sample collection and sequence data

We analysed 23 faecal samples from 20 lions (three lions were sampled twice, about six months apart). Shotgun short-read Illumina sequence data were generated from all samples (150bp reads, average 31.5m reads per sample), and Oxford Nanopore long-read data from 10 samples using version 9 chemistry (average 1.4m reads per sample, average length 2443, N50=4894).

### 2.2 Classification of the lion gut microbiome

**Fig. 1:**
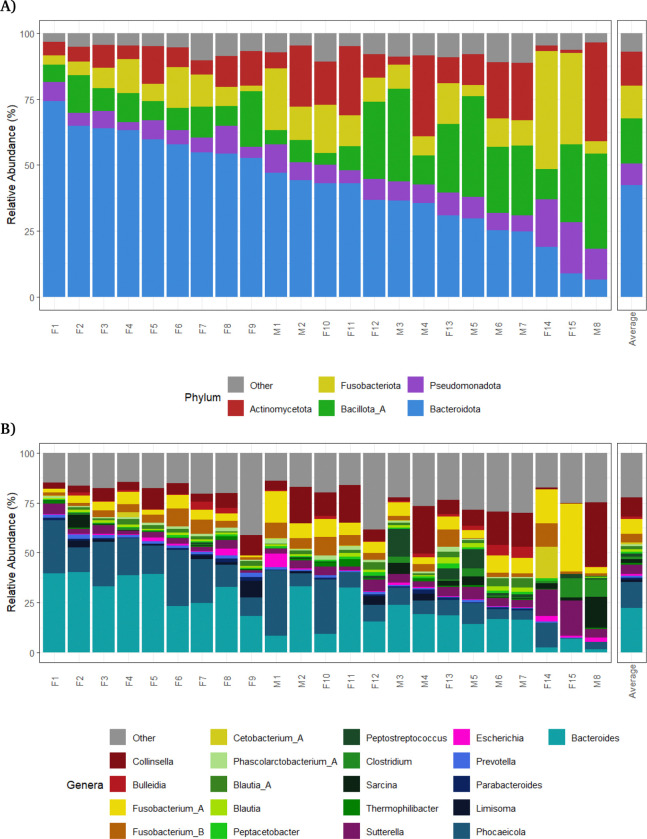
The lion gut microbiome is diverse and dominated by Bacteroides genera. Bar plot showing the relative abundance of **(A)** the top five bacterial phyla and **B)** the top 20 bacterial genera present in the *Panthera leo melanochaita* gut microbiome. Genera are coloured by phylum using the colour scheme shown in panel A. F = Female; M = Male

Kraken2 (v2.1.6) with Bracken (v3.0.1) was used to classify short-read sequences at the phylum and genus levels of the Genome Taxonomy Database (GTDB) (Fig. 1). The five phyla with the highest abundance, in order of abundance, were: Bacteroidota (42.4%), Bacillota A (17.1%), Actinomycetota (12.9%), Fusobacteriota (12.6%) and Pseudomonodota (8.1%) (Fig. 1A). The most abundant genera were *Bacteroides* (22.1%), *Phocaeicola* (13.3%), *Collinsella* (9.7%) and *Fusobacterium A* (7.4%) (Fig. 1B).

There was variability in the gut bacteria composition among the lions at genus level: eight lions had *Fusobacterium A* (F14 and F15), *Collinsella* (M4, M6 and M8), *Phocaeicola* (F6, M1 and F10) as the most abundant genera rather than *Bacteroides*. Of interest, lion F15 had recently given birth to cubs and lions F14 and M1 were the oldest lions sampled (10 years old).

### 2.3 Novel meta-genome assembled genomes from the lion gut microbiome

**Fig. 2:**
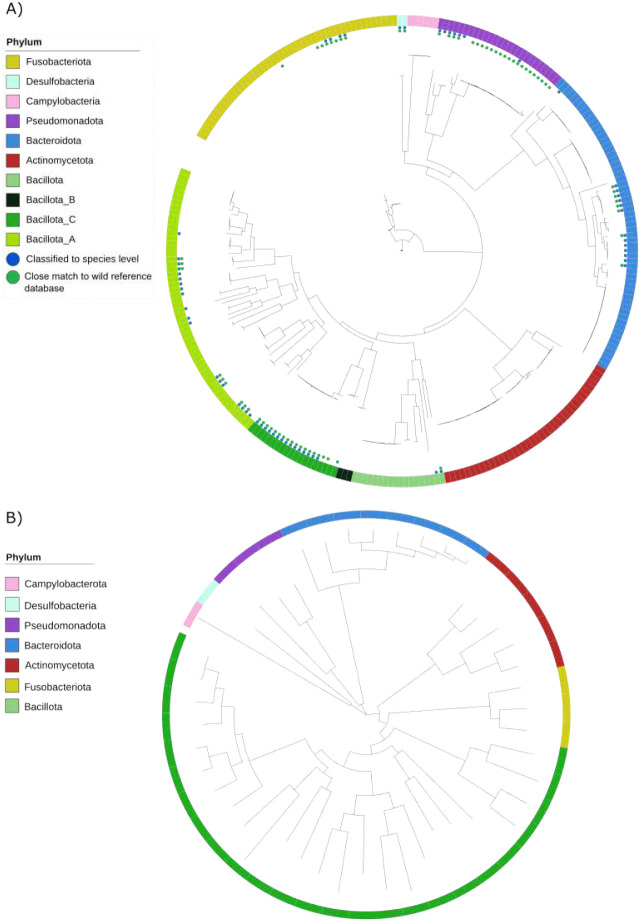
The majority of MAGs created from the lion gut represent novel species Phylogenetic trees showing the diversity of MAGs created using **A)** 272 short-reads and **B)** 46 long-reads of medium to high quality sequenced from the lion gut microbiome. MAGs from short-reads were compared to 5 596 MAGs from a wild animal reference database.

We constructed two sets of metagenome assembled genomes (MAGs). Using short-read data 272 medium and high-quality MAGs were constructed (Fig. 2A), and using long-read data (polished with short-read data), 46 medium and high-quality prokaryotic MAGs were built (Fig. 2B). They were classified using GTDB-tk (Chaumeil et al., 2022). In addition, 180 high-quality viral MAGs were generated using long-read sequences.

At the phylum level, 94 MAGs from short-reads (35%) were of the phylum Bacillota, 60 Bacteroidota (22%), 46 Fusobacteriota (17%), 38 Actinomycetota (14%), 26 Pseudomonadota (10%), six Campylobacterota (2%) and two Desulfobacterota (1%). At the genus level, 29 MAGs from short-reads were classified as *Collinsella* and 29 as *Fusobacterium B*. The list of MAGs from short-read data and associated metadata including GTDB classfication can be found as Supplementary Data SD1.

The novelty of the data was revealed when the MAGs from short-read data were compared to 5,596 reference genomes created from the gut microbiome of 180 wild animals (Youngblut et al., 2020). Of the 272 MAGs, 211 (78%) had less than 95% average nucleotide identity (ANI) with any reference MAGs (Fig. 2A).

Of the MAGs from long-read data, 25 were classified as Bacillota (54%), 8 as Bacteriodota (18%), three as Fusobacteriota (7%), five as Actinomycetota (11%), three as Pseuomonadota (7%) and one each as Campylobacterota and Desulfobacteriota. Only 20 could be classified at species level – 24 were classified at genus level and two at family level. The list of MAGs and associated metadata including GTDB classfication can be found as Supplementary Data SD2A.

We also constructed 108 high-quality viral MAGs, the vast majority being Caudoviricetes – only four were classified below class level, so the MAGs are likely to be highly novel. The list of viral MAGs with metadata can be found as Supplementary Data SD2B.

### 2.4 Supplementary classification

The high number of unclassified reads (24.5%) shows considerable novelty in the data, and different dominant taxa to previous research. The MAGs we constructed allowed us to improve classification and then studied the functional impact of the microbiota.

**Fig. 3:**
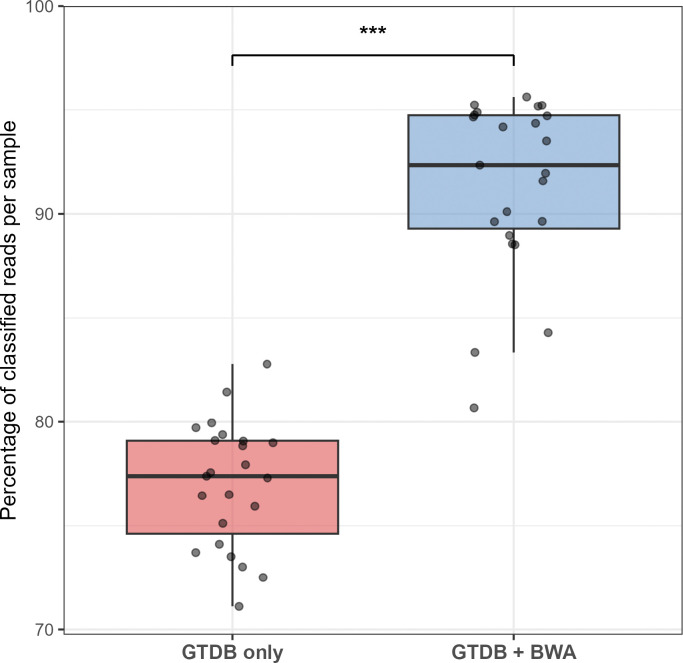
Inclusion of MAGs reduces the proportion of unclassified reads, while increasing assignment to known genera. Box plot showing the percentage of total reads classified at the genus level using Kraken2 with the Genome Taxonomy Database (GTDB) alone compared to GTDB supplemented with novel lion gut MAGs (via BWA mapping). *** = p <0.005

We aligned the remaining unclassified short-reads to the 318 MAGs created with long- and short-read data (Fig. 3), reducing the proportion of unclassified reads to 9.2% (an increase of 14.37%). This difference was significant using a paired t-test (p = 2 × 10^−16^).

### 2.5 Functional classification of the lion gut microbiome

**Fig. 4:**
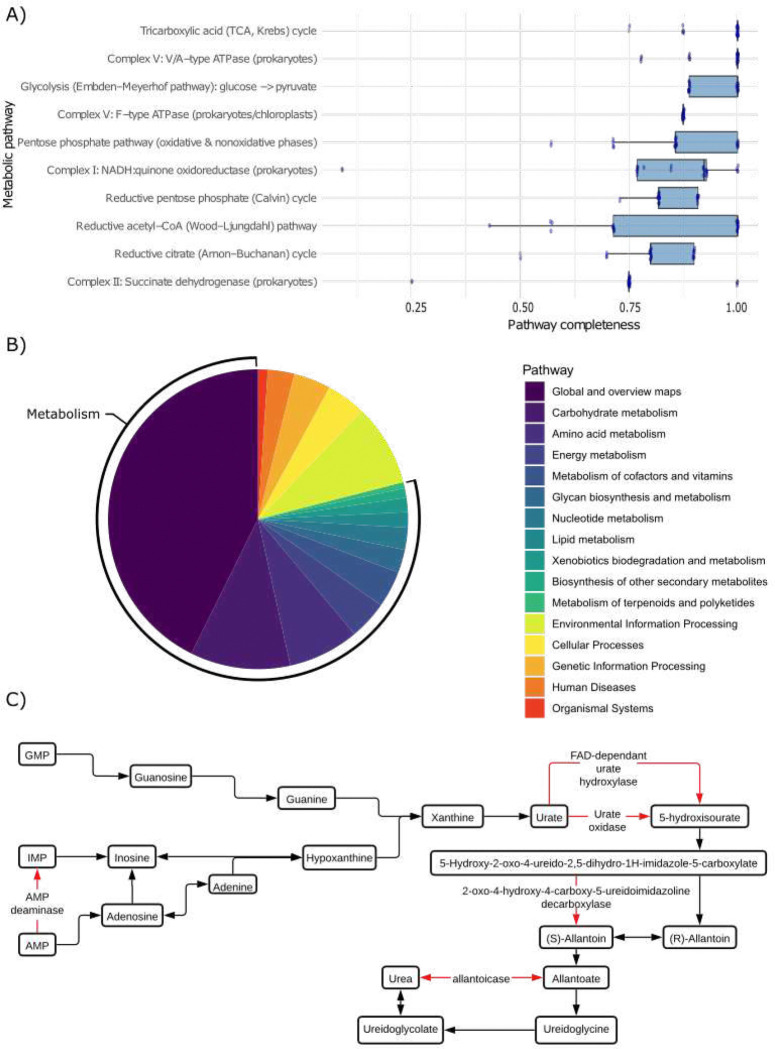
The lion gut microbiome has a large proportion of genes involved in amino acid and purine metabolism. **A)** Box plot showing the top 10 functional pathways with the average completeness across all lion gut microbiome samples. **B)** Pie chart showing the distribution of KEGG pathways detected across all samples. A pathway is counted as present if at least one protein hit to that KEGG pathway was identified in any sample. **C)** Overview of purine metabolism and urate degradation pathway. Red arrows indicate a gene not found in the 23 lion gut microbiomes.

To better understand the functionality of microorganisms in the lion gut, 27,916 short-reads were annotated using GHOSTkoala. We found 44.6% of reads were annotated to KEGG Orthologs (KO) and Pathways (Fig. 4). Of the successfully annotated genes, nearly 80% of those found in the lion gut microbiome were related to metabolism (Fig. 4A). Within the genes associated with metabolism, the most abundant pathway was global and overview maps (2,674 genes). Carbohydrate (676 genes), amino acid (479 genes) and energy (262 genes) metabolism were the second, third and fourth most abundant pathways, respectively. Outside of metabolism, environmental information processing (555 genes), genetic information processing (252 genes), and cellular processes (266 genes) had the highest abundances of associated genes.

Contigs created from short-reads were also compared against the KEGG database and analysed for pathways with highest average completeness across samples (Fig. 4B). The tricarboxylic acid (TCA, Krebs) cycle showed the highest mean completeness (0.97), followed closely by the Complex V: V/A-type ATPase (prokaryotes) (0.97) and glycolysis (Embden–Meyerhof pathway) (0.95). Other major energy-generating pathways were also near complete, including the Complex V: F-type ATPase (prokaryotes/chloroplasts) (0.88), pentose phosphate pathway (0.86), and Complex I: NADH:quinone oxidoreductase (prokaryotes) (0.85). Several carbon fixation and anaerobic energy metabolism pathways also displayed high completeness, such as the reductive pentose phosphate (Calvin) cycle (0.85), reductive acetyl-CoA (Wood–Ljungdahl) pathway (0.83), and the reductive citrate (Arnon–Buchanan) cycle (0.81). The Complex II: succinate dehydrogenase (prokaryotes) was also substantially represented (0.75).

Considering the high purine diet of lions, nucleotide metabolism (150 identified genes) was investigated more specifically. Under nucleotide metabolism, purine metabolism was analysed through guanine, adenine and urate degradation pathways (Fig. 4C). Metabolism of guanine monophosphate (GMP) and adenine monophosphate (AMP) could be entirely performed by the lion gut microbiome except for the conversion of urate to 5-hydroxyisourate. In this case, no proteins were annotated as either urate oxidase or flavin adenine dinucleotide (FAD)-dependant urate hydroxylase, suggesting that these enzymes are absent in the gut bacteria of lions.

### 2.6 Sex and season as causes of variability in the gut microbiome of lions

**Fig. 5:**
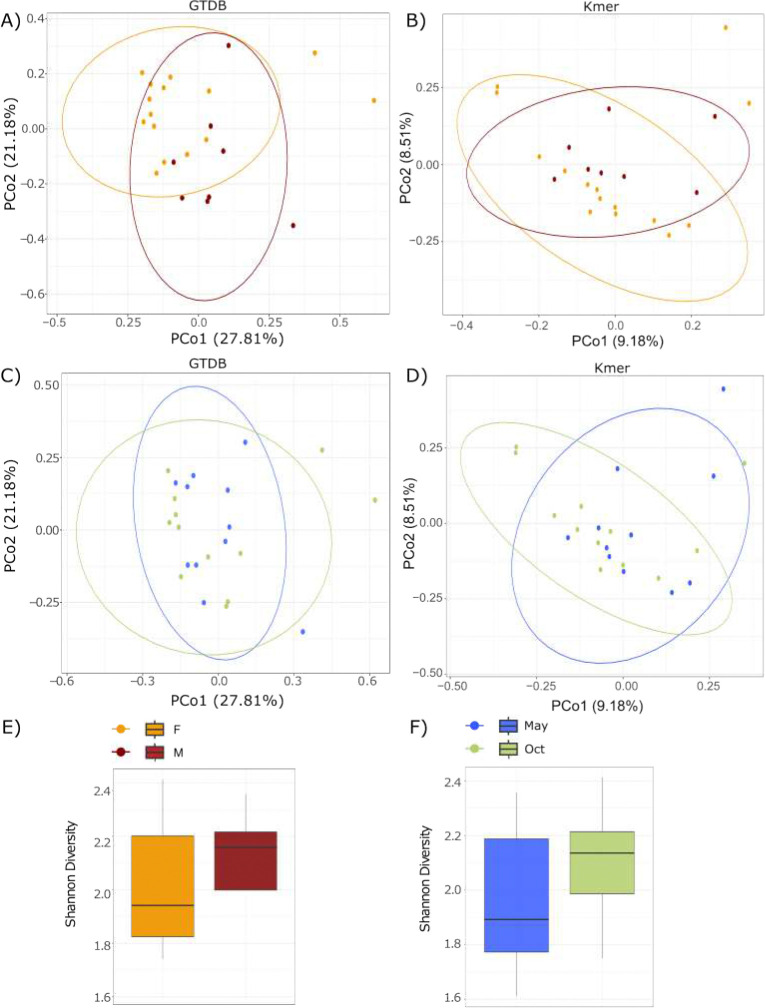
Sex and season explain little variance in the lion gut microbiome. Principal coordinates analysis (PCoA) plots the gut microbiome of male and female lions using **A)** weighted UniFrac distances of gut bacteria and **B)** k-mer distances. PCoA plots of gut microbiome of lions in May (winter) and October (summer) using **C)** weighted UniFrac distances of gut bacteria and **D)** k-mer distances. There was no significant difference between seasons or sexes. Shannon diversity of the gut microbiome of **E)** male and female lions and **F)** lions in May and October using species counts.

Using data from the Bracken genus level, the Bray-Curtis dissimilarity index was measured to identify whether the overall gut microbiome diversity of lions differed between males and females. After multiple hypothesis correction, there were no significant differences in the composition of the gut microbiome between male and female lions (*P* = 0.096; d.f. = 22)(Fig. 5A). Due to the large proportion of unclassified reads, a reference agnostic approach was used to confirm the findings. Sourmash was used to divide reads into k-mers and calculate Bray-Curtis distances between samples. The results confirmed that there were no significant differences in the composition of the gut microbiome between the sexes (*P* = 0.255; d.f. = 22)(Fig. 5B). However, at a more specific level, one species, *UMGS1663 sp012513065* was significantly more abundant in male lions (P = 0.0007) compared to female lions.

The Shannon diversity of the samples ranged from 1.74 to 2.4. There was no significant difference in Shannon diversity between the two sexes (*P*= 0.68; d.f. = 22) (Fig. 5E).

Similar analyses were performed to identify differences between lions caught in winter and summer. There was no significant difference in the lion gut microbiome between lions captured in either season (*P* = 0.44; d.f. = 22) (Fig.5C) using Bray Curtis dissimilarity. Once again, a reference agnostic approach confirmed this finding (*P* = 0.255; d.f. = 22) (Fig.5D). There was also no significant difference in Shannon diversity between lions captured in either season (*P* = 0.18; d.f. = 22) (Fig.5F). No bacterial species were significantly more or less abundant in lions captured in winter compared to those captured in summer.

## 3 Discussion

### 3.1 Classification of the lion gut microbiome

#### 3.1.1 Core taxa

On average, *Bacteroides* was the most abundant genus in the gut microbiome of free-living lions in Etosha National Park (22.1%). The majority of *Bacteroides* reads were classified as *Bacteroides sp. 900766005* (13.6%). There are three genomes for this species on the GTDB database, one created from metadata sampled from domestic cats (*Felis catus*), (PLAZA ONATE, 2023), the other two derived from human gut samples collected in China (Nayfach et al., 2019). *Bacteroides* is often the most abundant genus in the mammalian gastrointestinal tract and, in some studies, is the only genus common between herbivores, carnivores and omnivores (Wu et al., 2022). *Bacteroides* species are Gram-negative, obligate anaerobic, non endospore-forming bacilli that can be motile or non-motile (Karlsson et al., 2011). *Bacteroides* species are involved in various metabolic activities, including the degradation of complex carbohydrates and the production of short-chain fatty acids (SCFA) (Houtman et al., 2022). Indeed, humans with a high intake of protein and animal fat in their diet have a high abundance of *Bacteroides* (Wu et al., 2011). Similarly, a number of carnivores with high protein and fat diets have high abundances of *Bacteroides* (percentage abundances are mentioned in brackets), including black-backed jackals (*Canis mesomelas*) (15.1% (Menke et al., 2014) and 15.76% (Wu et al., 2022)), wild wolves (*Canis lupus*) (16 %) (Zhang and Chen, 2010), dholes (*Cuon alpinus*) (13.58%) (Wu et al., 2022) and raccoon dogs (*Nyctereutes procyonoides*) (36.91%) (Wu et al., 2022).

However, *Bacteroides* is not the most abundant genus in the gut of all carnivores. In the gut of spotted hyena (*Crocuta crocuta*) in Masai Mara National Reserve in Kenya, *Clostridium* (17.88%) was the most abundant genera and *Bacteroides* (3.38%) far less so (Rojas et al., 2022). Diet quality appears to be a major driver of these patterns; Rojas et al. (2022) demonstrated that *Bacteroides* abundance in hyenas increased fivefold during a severe two-year drought, when prey availability declined and hyenas were forced to scavenge more frequently and likely consumed a broader range of tissues. In contrast, during periods of high prey availability, *Clostridium* remained dominant (Rojas et al., 2022). Similarly, the gut of wild cheetah (*Acinonyx jubatus*) in central Namibia, is dominated by *Clostridium* (24.5% (Menke et al., 2014) and 19.5% (Wasimuddin et al., 2017)) while *Bacteroides* is comparatively low (5.4%). In the same paper, Wasimuddin et al. (2017) positively correlate the abundance of *Clostridium* species in the cheetah gut with captivity, suggesting the difference in diet as a possible cause of this difference, again supporting the idea of a diet as a core driver of microbiome diversity.

A notable distinction between lions and cheetahs is their social vs solitary natures; lions exhibit a cooperative, pride-based hunting strategy, whereas cheetahs typically hunt and consume prey individually (Hilborn et al., 2018). Other carnivores such as jackals, wolves, and dholes, which share a similar abundance of *Bacteroides* to lions, also engage in pack hunting (Barber-Meyer et al., 2016; Hayward and Kerley, 2008). This difference in social organisation could have a variety of effects on the gut microbiome. Firstly, pack-hunting animals are more likely to horizontally transfer bacterial species between individuals, especially in animals with a fluctuating pride structure, like lions (Sarkar et al., 2020). In contrast, solitary hunters have limited interactions with conspecifics, resulting in lower rates of bacterial exchange (Logan et al., 2010). Secondly, pack hunting and solitary hunting affect dietary patterns: solitary hunters consume the most nutritious portions of prey immediately, while pack hunters usually distribute resources based on social hierarchies (Hayward and Kerley, 2008). Finally, these different hunting tactics have different dietary requirements, for instance, cheetahs’ reliance on short bursts of energy necessitates a high protein intake, whereas lions prioritise a diet rich in both protein and fat (Hayward and Kerley, 2008). These differences affect the gut microbiome and together may be the reason for a higher abundance of *Bacteroides* in the gut of pack-hunters compared to solitary hunters. However, solitary hunting cannot be the only explanation for reduced abundance of *Bacteroides* in the carnivore gut as hyena also have low *Bacteroides* abundance, despite their large fission-fusion clans (Smith et al., 2007; Rojas et al., 2022). Taken together, current evidence suggests that dietary quality, prey composition, and tissue selection are likely the primary drivers of dominant bacterial genera in carnivore gut communities, with social structure also influencing microbiome diversity.

Differences between African and the captive, hybrid lions further illustrate the impact of ecological and environmental factors on gut microbial composition. The captive, hybrid and Asiatic lions exhibit *Fusobacterium* (35% and 14% respectively) as the most abundant genus in their gut (Mittal et al., 2020; Sun et al., 2025), although in the Asiatic lions, this abundance was diet dependant. Regardless, the high abundance of *Fusobacterium* is in contrast to our findings regarding the African lion (*Fusobacterium* was 7.4% abundant). To check whether taxonomic differences at genus/species levels could be due to updates in taxonomy and new classification approaches (Parks et al., 2018), we reclassified the sequences collected from the three lions in Mittal et al. (2020) using our own custom pipeline (the other lion studies discussed generated 16S rRNA gene data and so are not directly comparable). Although, we saw changes in the relative abundance of many genera compared to the original paper (Supplementary figure S1), there remained very large differences in bacterial genus and phyla abundance between the African and Asian subspecies. When reclassified through our own pipeline, the most abundant phylum in these captive hybrid lions was Firmicutes (Bacillota). The high abundance of Firmicutes aligns with more recent studies on the same subspecies also in captivity (Sun et al., 2025).

There are multiple possible reasons for the difference in bacterial gut genera between hybrid lions, Asiatic lions (*Panthera leo persica*) and African lions (*Panthera leo melanochaita*). Firstly, the gut microbiome is known to differ between hosts of different geographies, for example humans (Goertz et al., 2019) and some carnivores (Adams et al., 2021; Colborn et al., 2020). Secondly, the lions in our study were born in the wild and have been free roaming their entire lives, while the hybrid and Asiatic lions were captive and on controlled diets (Mittal et al., 2020). As discussed above, captivity and its related diet is a probably cause for at least some differences in the gut microbiome of these host subspecies. Finally, it is possible that changes in bacterial genera in the gut are caused by their allopatric separation over a prolonged period of evolutionary history (de Manuel et al., 2020). The two subspecies share a similar proportion of high-level taxa such as phylum but differ at lower levels such as genus caused by minor phylogeographic changes over time, supporting the idea of evolutionary divergence. Allopatric separation is a plausible explanation for the differences in abundance between the lions native to Namibia and India as they still retain some similar abundances of bacteria common to both subspecies. For example, both groups have a high abundance of *Phocaiecola*, which may be a conserved genus within the gut microbiome of lions for functional reasons, thus it is retained in similar abundance regardless of geography. On the other hand, genera such as *Bacteroides*, *Sutterella* and *Fusobacterium* were able to shift over time either because they perform similar functions within the lion gut or because they do not serve an essential function within the lion gut. Indeed, temporal changes in the gut microbiome of carnivores are well-documented (Schlomann and Parthasarathy, 2019), specific studies indicating substantial fluctuations in *Bacteroides* abundance over years (Rojas et al., 2022).

### 3.2 Novel meta-genome assembled genomes from the lion gut microbiome

The 318 newly generated bacterial MAGs newly generated from short-readd and long-read data and 102 new viral MAGs contribute to current wild animal databases and expand gut metagenome comparisons given the absence of prior sampling or sequencing of the African lion gut microbiome. Metagenomic analyses are negatively affected by incomplete databases (de la Cuesta-Zuluaga et al., 2020), which can lead to incorrect conclusions drawn about the wild animal gut microbiome and can affect diversity metrics, beta diversity comparisons and abundance charts. The MAGs generated in this study provide a significant contribution to current databases. Given that the majority of MAGs could not be classified to the species level and differ in average nucleotide identity (ANI) from current databases, they likely represent a variety of new species which could enrich current databases and aid in understanding the functions of the lion gut microbiome in the future.

In view of the fact that none of the MAGs in the Actinomycetota phylum were classified to the species level, it is likely that there are species in this phylum unique to the lion gut (Richter and Rosselló-Móra, 2009). Interestingly, the phylogenetic distances between MAGs in this phylum were noticeably lower than other phyla, which could signify genomes from one or two novel species unique to the lion gut. A similar pattern of several closely related MAGs not classified to the species level was identified in a few phyla, including, Bacillota C, Bacteroidota, Bacillota B and Pseudomonadota. While all 272 MAGs from short-reads and 46 MAGs from long-reads are unlikely to represent new species, we envisage MAGs from multiple new species identified from this research. At the genus level, a large proportion of MAGs were classified as *Collinsella* and *Fusobacterium B*, implying a similar addition to these genera in current databases.

### 3.3 Functional classification of the lion gut microbiome

Functional analysis showed metabolism as the most abundant pathway represented in the lion gut microbiome, with carbohydrate and amino acid metabolism in highest abundance besides the generalised global and overview maps. Bacteria in the carnivore gut have a similar proportion of amino acid metabolism and carbohydrate metabolism genes (Zhu et al., 2018). This discovery of high carbohydrate metabolism is not necessarily surprising as carbohydrates are common in the lion’s diet (Borstlap, 2002). Carbohydrates can be ingested from prey tissues and plant matter consumed incidentally during feeding. The abundance of carbohydrate-metabolising genes suggests that bacteria in the lion gut have the metabolic potential to process incidental carbohydrate intake (e.g. fresh grass incidentally consumed, or ungulate stomach contents).

Aerobic-respiratory signatures are very well represented in the lion gut: the tricarboxylic acid (TCA) (Krebs) cycle along with components of the electron transport chain (Complex I, Complex II) and ATP synthase (Complex V) pathways scored among pathways the highest average completion of genes. The presence of both F-type and V/A-type ATPases is particularly notable: while F-type ATPases typically drive ATP synthesis via the proton-motive force, V/A-type ATPases are reversible machines, and in gut bacteria may function using ATP hydrolysis to pump protons out of the cell in order to maintain cytoplasmic pH under acid, anaerobic or stress conditions (Zubareva et al., 2020). The ability to maintain a consistent pH is essential for bacteria in the physiologically stressful environment of the carnivore gastrointestinal tract (Barathan et al., 2024). In parallel, the glycolysis (Embden–Meyerhof) pathway and the pentose phosphate pathway also show high completeness. Both pathways are core aerobic and anaerobic routes of carbohydrate catabolism (Gupta and Gupta, 2021). Their high completeness aligns with the overall enrichment of genes related to metabolism and respiration observed across the lion gut MAGs.

Strongly anaerobic and autotrophic pathways are also well represented: the reductive acetyl-CoA (Wood–Ljungdahl) pathway and the reductive TCA (Arnon–Buchanan) cycle indicate a capacity for acetogenesis, CO_2_ fixation, or hydrogen-coupled metabolism typical of strictly anaerobic bacteria (Ragsdale and Pierce, 2008). Finally, the Calvin (reductive pentose-phosphate) cycle was highly complete in most samples which is interesting as this cycle is present in less than 7% of microbial genomes (Asplund-Samuelsson and Hudson, 2021). Although traditionally associated with photosynthetic carbon fixation, in gut bacteria the pathway (or RuBisCO-like enzyme variants) may participate in CO_2_ refixation, redox balancing, or nucleotide salvage rather than canonical photosynthesis (Asplund-Samuelsson and Hudson, 2021).

Taken together, these data emphasize that the lion gut microbiome is not simply composed of obligate anaerobic meat-degraders but harbours a functionally versatile community, one able to operate robust aerobic respiration, facultative fermentation, anaerobic autotrophy/acetogenesis, ion-gradient maintenance and redox recycling. Such metabolic flexibility likely reflects adaptation to the protein-rich, and intermittently oxygenated gut environment of a carnivore.

Furthermore, bacteria in the lion’s gut had all the genes necessary to metabolize purines to urea independently of the host, except for one step, namely, the conversion of urate to 5-hydroxyisourate; none of the reads corresponded to the urate oxidase gene or FAD-dependent urate hydrolase. We focused on this pathway because of lions’ high purine diet (Herring et al., 2021). Purine metabolism is essential for many carnivores, including lions, to effectively utilise dietary resources and maintain metabolic homeostasis (Zhu et al., 2018). Purines are essential components of nucleic acids and play vital roles in cellular processes, making their metabolism crucial for cellular function and energy metabolism. The presence of 18 proteins involved in purine metabolism indicates that microorganisms in the lion gut are actively assisting lions to digest important components of their diet.

### 3.4 Sex and season as causes of variability in the gut microbiome of lions

We found no significant differences in overall classification, read composition or alpha diversity in the gut microbiome between sexes or seasons. Initially, a difference in the composition of the intestinal microbiome between sexes would be expected, as this difference has been discovered in a few wild species, including minks (*Neovision vision*)(Lafferty et al., 2022), western lowland gorillas (*Gorilla gorilla gorilla*)(Pafčo et al., 2019), dholes (*Cuon alpinus*)(Wu et al., 2016). In the cases where sex drives a difference in gut microbiome composition, differences are often hypothesised to arise from behavioural or ecological distinctions between males and females, particularly consistent differences in diet, such is the case with foragine gorillas (Pafčo et al., 2019). However, other studies have not identified a difference between sexes in gut microbiome composition and alpha diversity in animals such as cheetahs (*Acinonyx jubatus*) (Wasimuddin et al., 2017), Egyptian mongooses (*Herpestes ichneumon*) and chimpanzees (*Pan troglodytes schweinfurthii*)(Degnan et al., 2012). While male and female lions can differ in diet due to their hierarchical feeding habits, pride compositions of lions are more complex than simple hierarchy; all-female prides are common, prides are often split into smaller groups, cooperative hunting is not always preferred (many solitary hunters exist) and sometimes pride hierarchies are not apparent (Rubenstein and Wrangham, 2014). The flexible nature of lion prides is likely to cause fluctuating dietary overlap and frequent interactions between individuals, promoting horizontal transfer of gut microorganisms. Variable social structures could therefore homogenize gut microbial communities between males and females, reducing or eliminating sex-linked microbiome differences.

Based on simulated PERMANOVA analyses, approximately 10 subjects per group – similar to our sample size — have about 90% power to detect a relatively strong community-level effect (*ω*^2^ ≈ 0*.*02) (Kelly et al., 2015). Smaller differences (below this threshold) would likely go undetected at our current sample size. Thus, while our dataset is adequately powered to identify major sex- and season-related shifts in community composition, it may lack sensitivity to more subtle or taxon-specific effects.

## 4 Conclusion

This study represents the first comprehensive taxonomix analysis of the microbiome classification of African lions (*Panthera leo melanochaita*) and reconstructing the largest collection of metagenome-assembled genomes (MAGs) from lion faecal samples and substantially expanding reference genome representation for this host in public databases. The most abundant bacterial genera present in the 23 African lion gut microbiome samples were *Bacteroides* and *Phocaeicola*, two genetically related genera from the same family. The high abundance of *Bacteroides* in the lion gut was in contrast to the only previous study investigating lions specifically, which found *Fusobacterium* to be the most abundant genera in the gut microbiome of three captive, hybrid lion (Mittal et al., 2020). The differences may be due to captivity experiences of the hybrid lions or allopatric separation between the two subspecies preventing transfer of microbial species and allowing for changes in the gut microbiome composition over time. Furthermore, we believe that since the genus *Phocaiecola* was similarly abundant in both subspecies, it serves a conserved function in the lion gut microbiome.

This study identified 272 MAGs from short-reads and 46 MAGs from long-reads which will contribute significantly to current databases. Supplementing current databases with these MAGs decreased the percentage of total unclassified reads from 24. 51% to 9.24% indicating a noticeable contribution to classification efforts. Given that the majority of MAGs could not be classified to the species level and differed in average nucleotide identity by more than 95% from current databases, they represent new species not present in current databases. Further analysis of these MAGs will help to identify specifics about these new species and support the development of current databases.

Finally, functional classification revealed the lion gut microbiome to have a high abundance of metabolic genes. Specifically, purine metabolism was well-represented, indicating commensal microorganisms assisting the lions to digest important components of their diet. We found the gut microbiome did not differ significantly between male and female lions nor did it differ between lions caught in winter or summer.

## 5 Methods

### 5.1 Study area

**Fig. 6:**
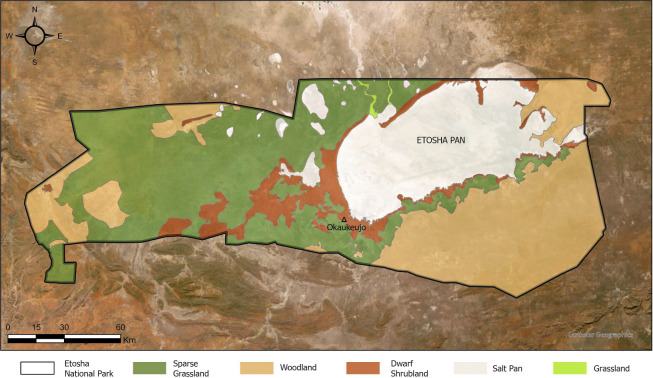
Etosha National Park vegetation structure. The black line represents the boarders of the park. The triangle marks Okaukuejo camp.

Faecal samples were collected from free-living lions in Etosha National Park (Fig. 6). The park is a 22 270 km^2^ fenced reserve in northern Namibia. Etosha is subdivided into three major habitats; woodlands on the far west and east of the park, open grassland plains in the centre and a hypersaline pan in the central region (Heydinger and Packer, 2022). The park experiences approximately 350–460mm of rainfall per year (Turner et al., 2022), with December to March being the hottest and wettest months (Turner et al., 2022). In winter, temperatures range from 18–28°C during the day, while below zero temperatures are common at night. In the summer, temperatures range from 20–34°C during the day (Turner et al., 2022). The sampled lions primarily occupied the plains area surrounding Okaukuejo (19.175°S, 15.924°E).

### 5.2 Sampling

This microbiome project was part of a larger carnivore project in a collaboration between the University of the Witwatersrand, the Leibniz Institute for Zoo and Wildlife Research and the Etosha Ecological Institute. The study lions were sedated for a maximum of 10 minutes to fit or remove tracking collars and collect health status information, and fresh faecal samples. A total of 23 faecal samples were collected in OMNIgen GUT tubes (DNAGenotek, Kanata, Canada) from 20 lions, across 12 prides (Supplementary table S1). Immobilisation of the lions took place in two seasons, namely winter (May 2022) and summer (October 2022). Samples were placed in a cooler box at 15 °C within 5 minutes of collection and then moved to a −20°C freezer within 24 hours for longer term storage.

### 5.3 DNA extraction and sequencing

After the final samples were collected in October 2022, genomic DNA was extracted from 250 milligrams of each faecal sample using the DNeasy PowerSoil extraction kit (QIAGEN, Hilden, Germany) following the manufacturer’s instructions. DNA was then shipped to South Africa.

Illumina short-read shotgun sequencing was done at National Institute for Communicable Diseases in Johannesburg, South Africa: DNA preparation was performed using the Illumina DNA prep kit and PCR was performed using the Illumina DNA prep reference guide (Illumina, 2024). The samples were sequenced on the Illumina Nextseq 2000 platform. Reads were provided in FASTQ format (average of 30.4m reads per sample).

For long-read sequencing, 10 high quality samples were sent to the Genomics Platform of the South African Medical Research Council (SAMRC) for Oxford Nanopore Technology (ONT) sequencing. Long-read sequencing was performed on an Oxford Nanopore GridION platform with MinION R9.4.1 flow cells and SQK-LSK109 library preparation chemistry, yielding an average of 1.4 million reads per sample (N50 = 4,894). ONT data was provided in FAST5 format, which were converted into Pod5 format using the POD5 Python Package (Oxford Nanopore, 2024). Base calling was done using Dorado v.0.8.0 (Nanopore, 2024).

### 5.4 Bioinformatics analysis

#### 5.4.1 Short-read sequencing

**Fig. 7:**
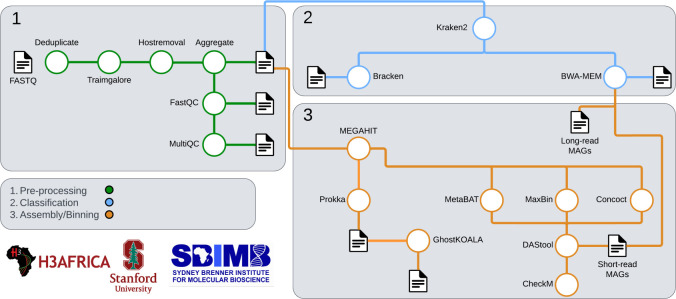
Nextflow pipeline used to process DNA reads created from the gut microbiome of lions in Etosha National Park, Namibia. Box 1 shows the steps for pre-processing the samples for removal of low quality reads and quality report generation. Box 2 shows the steps used for taxonomically classifying the reads. Box 3 shows the steps for assembling and binning the reads into contigs and Metagenome Assembled Genomes (MAGs).

The github.com/bhattlab/AWIGen2Microbome pipeline was used as a basis for processing the short-read metagenomic sequencing. The repurposed pipeline is shown in Fig. 7.

Short-reads reads were preprocessed by removing duplicate and low-quality reads. Trimgalore v0.6.7 (Krueger, 2015) parameters were set to a read quality (Phred score) greater than 30 and a minimum read length of 60 base pairs. Lion sequences were also removed from the samples by aligning them to a lion reference genome from Ensembl using Burrows-Wheeler Aligner Maximal Exact Matches (BWA-MEM) v0.7.17 (Li and Li, 2020). The quality of reads was then assessed using FastQC v0.11.9 and MultiQC v1.13 (Ewels et al., 2016). After quality filtering and removal of orphan reads, samples contained an average of 4.9 million reads per sample.

Preprocessed short-reads were classified using Kraken2 (Wood et al., 2019) with a *k*-mer length of 31 and read length of 130 against the Genome Taxonomy Database (GTDB version 226) and the April 2025 version of Kraken viral database, using the pre-computed index from https://benlangmead.github.io/aws-indexes/k2) followed by Bracken (Lu et al., 2017) for an estimation of relative abundance. Statistical analyses were performed using R v4.1.2. Taxonomic alpha and beta diversity were calculated with vegan v2.6–4. Linear regression for differential abundance of taxa, principal coordinate analyses were carried out using the R base package. PERMANOVA was used to test for significant differences in MDS and t-tests for differences in alpha diversity (Li et al., 2022). Phylogenetic trees were visualised with iTOL v655 (Letunic and Bork, 2007). Beta diversity was quantified and visualised using Bray-Curtis dissimilarity index. BrayCurtis dissimilarity was first quantified using Bracken classification, however, due to the large abundance of unclassified reads, a reference agnostic approach was performed using Sourmash v (Moore et al., 2022) to confirm the results for beta diversity measurements. A linear regression model was used to determine the relationship between bacterial genera and the sex of the lion or season the sample was collected.

Pre-processed short-reads were assembled into MAGs. Contigs with a completeness of 50% and above and contamination below 10% were retained for further analysis. Contigs were then binned into groups based on similarity using MetaBAT2 v2.15 (Kang et al., 2019), MaxBin v2.2.7 (Wu et al., 2016) and Concoct v1.1.0 (Alneberg et al., 2013) and then combined using DAStool v1.1.6 (Sieber et al., 2018). The following parameters were used:

- MaxBin – contig length ≥ 1 000, marker gene sets=107, probability threshold=0.9;
- MetaBAT – contig length ≥ 2 500, ≥95% contig quality for each bin, bin size≥200 000; and
- Concoct – k-mer length 4, contig length≥1 000.

MAGs were taxonomically classified using GTDK-tk v2.4.1 (Chaumeil et al., 2022). Lastly, MAG functional analysis was performed using Prodigal v2.6.3 (Hyatt et al., 2010), GhostKOALA (Kanehisa et al., 2016) and DRAM (Shaffer et al., 2020) v2.0.0-beta11.

#### 5.4.2 Long-read analysis

MAGs created from long-reads were assembled using OPERA-MS v0.9 in hybrid mode (Bertrand et al., 2019). SemiBin2 v2.2 (Pan et al., 2023) was used for binning of bacterial MAGs which were filtered using CheckM v1.2.2 Shklovsky et al. (2023) and GUNC v1.0.6 (Orakov et al., 2021) with threshold values of >50% completeness and <10% contamination and clade separation scores (CSS) < 0*.*4. Finally the MAGs were polished using short-read data and Polypolish v0.6 (Bouras et al., 2024) and dereplicated using dRep v3.5 (Olm et al., 2017). Prokaryotic MAGs were classified using the GTDB v226 database and GTDB-tk v2.4.1 (Chaumeil et al., 2022).

Given the high level of unclassified reads in our initial Kraken classification, we used the new MAGs that had been classified in a second phase of classification. Ideally, we would have built a custom Kraken database. Preliminary experimentation showed that the GTDB database was a better classifier on our data than the Kraken core_nt. The pre-computed GTDB index for Kraken cannot be used as a basis for a custom database, and it was computationally infeasible for us to build a custom database from GTDB v226 plus our new MAGs. A BWA-MEM2 index was constructed of the MAGs, and using a BWA-MEM2 version 2.2.1 (Li and Li, 2020), any reads that had not been mapped by Kraken which mapped to one of the MAGs with an AS flag of at least 140 (93%) was classified using MAGs classification. The disadvantage of this approach is that Bracken cannot be used.

Viral MAGs were assembled, polished and dereplicated in the same way (they were not binned as with relatively short genome size and the use of long-read data we believed that the advantages of binning were outweighed by risks of mis-assembly). We initially generated several hundred MAGs and so focussed only on very high quality MAGs. We used CheckV (Nayfach et al., 2021) as an initial quality check and chose only high-quality matches (≥ 90% completeness, ≤5% contamination). To further validate the viral MAGs, we mapped all the short-read data to the viral MAGS using BWA-MEM2 v2.2.1 (Li and Li, 2020) and classified support of a MAG as *high quality* if the short-read data of an individual sample gave > 80% coverage at a depth > 5, and as medium quality if the coverage was > 40% at a depth > 2, and otherwise as *not supported*. A viral MAG was accepted if it had high-quality validation from at least two samples, or high quality validation from one sample and three medium-quality validations. We then used geNomad (Camargo et al., 2024) and required *virus_score* ≥ 0*.*9, at least 3 hallmark genes, at least 90% of the MAG was covered by the short or long-read data for that sample (according to geNomad) and a marker enrichment score of at least 10.

Since we were only able to classify most of the viral MAGs at class level, we tried three approaches. We report the taxa given by geNomad. We also tested using Kraken 2’s viral database, which yielded the same result. We also BLASTed the MAGs against the the JGI IMG_VR_2022–12-19_7.1 database (Camargo et al., 2022), requiring an 80% identity. identity and coverage of at least 50%. The class classifications were consistent but we were not able to find any match to a specific entry in the database witg > 80% identity.

#### 5.4.3 Supplementary classification of short-reads

Short-reads not classified using Kraken2 were extracted and classified using BWA-MEM v2.2.1 (Li and Li, 2020) against medium- and high-quality MAGs created from long- and short-read sequences.

## Supplementary Material

Supplement 1

Supplementary Files

This is a list of supplementary files associated with this preprint. Click to download.

• Supplementaryfiguresandtables.pdf

## Data Availability

The datasets supporting the conclusion of this article are available: in the European Nucleotide Archive as study PRJEB106514 https://www.ebi.ac.uk/ena/browser/view/PRJEB106514 (data generated by us); comparator lions from Mittal et al. (2020) can be found using NCBI SRA database with the Accession ID SRR9943707–83 under the BioProject ID PRJNA559605; and fasta files for the 5,596 wild gut microbiome comparator MAGs from Youngblut et al. (2020) can be found at http://ftp.tue.mpg.de/ebio/projects/animal_gut_metagenome_assembly/. The code use in analysis can be found at https://github.com/bhattlab/AWIGen2Microbiome and https://github.com/SBIMB/lion-mb.

## Footnotes

1 We use *Panthera leo persica* and ‘African-Asian hybrid lion’ in this paper as this is how the cited paper describes them; however, this subspecies is no longer considered distinct from *Panthera leo leo* by the IUCN Cat Classificaton Task Force.
