# Supplementary material for: The Gut Microbiome Profile of Lions in Etosha National Park, Namibia
Source: Res Sq. 2026 May 6:rs.3.rs-9092464. Preprint. [Version 1] doi: 10.21203/rs.3.rs-9092464/v1 (PMC13174769; doi:10.21203/rs.3.rs-9092464/v1)
Supplement: Supplement 1 [file NIHPPRS9092464V1-supplement-1.pdf]

6 Supplementary Material

Table S1: Demographic data for 20 immobilised lions. Summary of the number of faecal samples collected from lions in Etosha National Park, Namibia.

	winter (May)[#]	summer (October)[#]	Total
	11	12	20
Sex			
Male	5	3	6
Female	6	9	14
Age			
3-4 years	2	1	3
5-8 years	4	7	10
9-10 years	2	2	3
Unknown	3	2	4

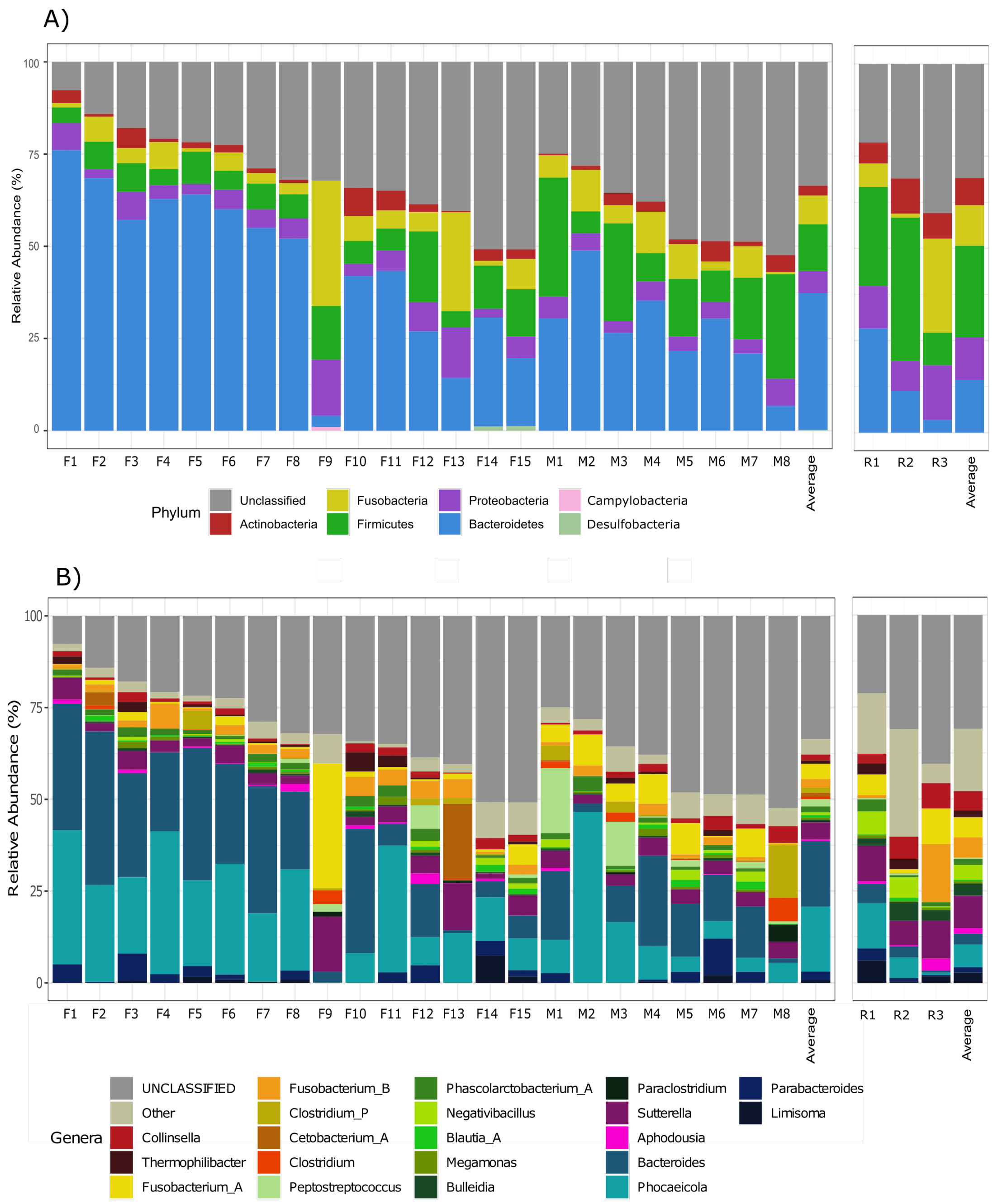

Fig. S1: Conversions from SGB to GTDB database results in changes in taxon abundance at the phylum and genus level. A) Bar plot showing the relative abundance of bacterial phyla present in the Panthera leo gut microbiome from MetaPhlAn classification converted to GTDB taxonomy. **B)** Bar plot showing the relative abundance of the top 20 bacterial genera present in the Panthera leo gut microbiome from MetaPhlAn classification converted to GTDB taxonomy. The left block shows free-living lions darted in Namibia for this study while the right block shows three reference lions from India (Mittal et al., 2020). Genera are coloured by phylum using the colours shown in panel A. F = female; M = Male; R = reference lion; SGB = species level genome bin; GTDB = genome taxonomy database
